# Myeloma Cells Down‐Regulate Adiponectin in Bone Marrow Adipocytes Via TNF‐Alpha

**DOI:** 10.1002/jbmr.3951

**Published:** 2020-01-16

**Authors:** Emma V Morris, Karla J Suchacki, Joseph Hocking, Rachel Cartwright, Aneka Sowman, Beatriz Gamez, Ryan Lea, Matthew T Drake, William P Cawthorn, Claire M Edwards

**Affiliations:** ^1^ Nuffield Department of Surgical Sciences University of Oxford Oxford UK; ^2^ NIHR Oxford BRC Oxford UK; ^3^ Oxford Centre for Translational Myeloma Research Oxford UK; ^4^ University/British Heart Foundation Centre for Cardiovascular Science The Queen's Medical Research Institute, University of Edinburgh Edinburgh UK; ^5^ Nuffield Department of Orthopaedics, Rheumatology, and Musculoskeletal Sciences Oxford UK; ^6^ Kogod Center on Aging and Division of Endocrinology Mayo Clinic College of Medicine and Science Rochester MN USA

**Keywords:** ADIPOCYTE, ADIPONECTIN, BONE MARROW ADIPOSE TISSUE, CANCER, MULTIPLE MYELOMA

## Abstract

Multiple myeloma is caused by abnormal plasma cells that accumulate in the bone marrow and interact with resident cells of the bone microenvironment to drive disease progression and development of an osteolytic bone disease. Bone marrow adipocytes (BMAds) are emerging as having important endocrine functions that can support myeloma cell growth and survival. However, how BMAds respond to infiltrating tumor cells remains poorly understood. Using the C57BL/KaLwRij murine model of myeloma, bone marrow adiposity was found to be increased in early stage myeloma with BMAds localizing along the tumor‐bone interface at later stages of disease. Myeloma cells were found to uptake BMAd‐derived lipids in vitro and in vivo, although lipid uptake was not associated with the ability of BMAds to promote myeloma cell growth and survival. However, BMAd‐derived factors were found to increase myeloma cell migration, viability, and the evasion of apoptosis. BMAds are a major source of adiponectin, which is known to be myeloma‐suppressive. Myeloma cells were found to downregulate adiponectin specifically in a model of BMAds but not in white adipocytes. The ability of myeloma cells to downregulate adiponectin was dependent at least in part on TNF‐α. Collectively our data support the link between increased bone marrow adiposity and myeloma progression. By demonstrating how TNF‐α downregulates BMAd‐derived adiponectin, we reveal a new mechanism by which myeloma cells alter the bone microenvironment to support disease progression. © 2019 The Authors. *Journal of Bone and Mineral Research* published by American Society for Bone and Mineral Research.

## Introduction

Multiple myeloma is an incurable hematological malignancy associated with clonal expansion of plasma cells within the bone marrow and the development of lytic bone disease. For many years it has been recognized that the success of myeloma cells to thrive within the bone marrow is in part due to their ability to interact favorably with the bone microenvironment. Myeloma cells can influence their neighboring bone cells, resulting in bone destruction and the release of growth factors into the local environment that can be utilized by the myeloma cells for growth and survival, a process often referred to as the vicious cycle.^1^ However, the influence that myeloma cells have on other non‐bone resident cells in the bone marrow is less clear. One of the most abundant cell types in the bone marrow is the adipocyte; these cells account for up to 70% of bone marrow volume in adult humans.^2^ Once thought of as inert space‐filling cells, adipocytes are now recognized as having specialized functions.^3^ These cells not only store energy in the form of triglycerides but also secrete important adipokines such as leptin and adiponectin. For many decades, bone marrow adipocytes (BMAds) were very much understudied and underappreciated. However, in recent years, this has changed, as their roles in metabolism^4^ and cancer have started to emerge. BMAds have been implicated in cancers that metastasize to bone, such as prostate^5^, ^6^ and breast cancer,^7^ as well as cancers that first become established in bone, such as acute myeloid leukemia^8^ and myeloma,^9^, ^10^, ^11^, ^12^, ^13^ where they appear to have a supporting role in fueling cancer growth, progression and bone disease. However, the mechanisms driving this relationship remain unclear.

BMAds are thought to contribute to bone homeostasis via the bioactive molecules they secrete, leading to the activation of numerous signaling pathways in many different cell types by means of autocrine, paracrine, and endocrine signaling.^14^ BMAds differ in their secretory profiles from both white and brown adipocytes. Furthermore, they have been shown to upregulate different genes when in close proximity to prostate cancer cells in comparison to subcutaneous adipocytes, suggesting that they have bone‐specific effects.^5^ Adiponectin is the most prevalent adipokine secreted by adipocytes. Although adiponectin is secreted by all adipose cells, it is most highly secreted by BMAds.^4^, ^15^ Adiponectin is known to play an important role in insulin sensitivity and fatty acid oxidation and has been shown to have antitumor effects.^16^, ^17^, ^18^ Adiponectin is paradoxically decreased during conditions of elevated adiposity, and our previous studies have implicated both obesity and hypoadiponectinemia in the progression of multiple myeloma and its associated bone disease.^19^, ^20^, ^21^ In the present study, we have investigated how myeloma cells alter BMAd biology to circumvent the tumor‐suppressive effect of adiponectin.

## Materials and Methods

### Animal model

Animal experiments were undertaken under UK Home Office Project Licenses 30/2996 and PCCCC8952. Animals were housed in individually ventilated cages in the Department of Biomedical Services, University of Oxford, with access to normal chow and water *ad libitum*. Studies were conducted using age‐, sex‐, and weight‐matched KaLwRij mice (Harlan Netherlands, Horst, the Netherlands). Because of the nature of the model, the speed of tumor development can vary between individual experiments. As such, “low” and “high” tumor burden, indicating less than an average of 10% myeloma cells in bone marrow or greater than an average of 30% myeloma cells in bone marrow, respectively, was used to describe the experimental conditions, rather than days after inoculation. Mice were inoculated with 10^6^ 5TGM1‐GFP cells or PBS vehicle control by intravenous injection. Mice were euthanized on day 16 to represent “low” tumor burden and day 23 to represent “high” tumor burden post‐inoculation (5 controls, 7 low‐tumor‐burden, and 6 high‐tumor‐burden mice were used; all animals were 12 weeks of age). Investigators were not blinded throughout the experiment. In separate experiments, animals used for osmium tetroxide analysis were inoculated as above and culled on day 24 when tumor burden was less than 10%, representing a “low” tumor burden model (4 controls and 5 inoculated; all animals were 8 weeks of age). Investigators were blinded at endpoint. Animals used for bone marrow plug analysis were inoculated as above and culled on day 23 (3 inoculated; animals were 6 months of age to ensure adequate visualization of BMAds and all mice had detectable myeloma). To investigate the effect of the JJN‐3 human cell line in vivo, age‐, sex‐, and weight‐matched Nod/Scid γ (NSG, NOD.Cg‐Prkdcscid Il2rgtm1Wjl/SzJ) mice were purchased from Charles River (Wilmington, MA, USA). Mice were inoculated with 10^6^ JJN‐3 cells or PBS vehicle control by intravenous injection. Animals were euthanized on day 23 (5 controls and 7 inoculated; all animals were 8 weeks of age). Investigators were not blinded throughout the experiment.

### Immunofluorescence

Sections from paraffin wax–embedded tissue samples were dewaxed, and antigen retrieval was performed using citric acid buffer. Sections were incubated with 1:200 chicken anti‐GFP (Invitrogen, Carlsbad, CA, USA; A10262) and 1:100 rabbit anti‐perilipin (Cell Signaling Technology, Danvers, MA, USA; 3470S) in 1% BSA/5% serum/phosphate buffered saline (PBS) overnight at 4°C. Sections were subsequently incubated for 1 hour at room temperature with 1:200 Alexa Fluor 488 anti‐chicken (Invitrogen A11039) and Alexa Fluor 568 anti‐rabbit secondary antibodies (Invitrogen A11011) and 1:5000 DAPI. Lipid uptake studies using patient samples were performed by layering the bone marrow sample over histopaque and centrifuging at 400*g* for 30 minutes. The fatty top layer of the sample was then removed and diluted 1:2 with normal media, and myeloma cells were then added for 24 hours before staining. The lipid uptake studies using cell lines were performed by liberating the lipids from ST2‐derived BMAds and incubating them with myeloma cells for 24 hours. Cells were then stained with 1:1000 BODIPY (Thermo Fisher Scientific, Waltham, MA, USA; D3922) or 1:500 Lipidtox (Thermo Fisher Scientific H24476) and 1:2000 Hoechst (Sigma, St. Louis, MO, USA; B2261) and visualized by confocal microscopy.

### Bone marrow plugs

Marrow plugs were prepared using femurs taken from 6‐month‐old mice that had been inoculated with 5TGM1‐GFP cells for 23 days. Marrow plugs were extracted and fixed on slides.^22^ The samples were then washed with PBS and stained using 1:500 Lipidtox (Thermo Fisher Scientific H24476) for 30 minutes.

### Quantification of tissue sections

Long bones were formalin‐fixed, decalcified in 14% EDTA, paraffin‐embedded, and sectioned along the mid‐sagittal plane in 4‐μm‐thick sections before staining with hematoxylin and eosin. Histomorphometric analysis was performed to quantify BMAd number and tumor burden using Osteomeasure histomorphometry software as previously described^23^ or ImageJ software.

### Osmium tetroxide staining

Formalin‐fixed, decalcified tibias were washed in Sorensen's phosphate buffer (81 mM KH2PO4, 19 mM Na2HPO4 · 7H2O, pH 7.4) and stained with 1% osmium tetroxide solution (2% w/v; Agar Scientific, Stansted, UK; diluted 1:1 in Sorensen's phosphate buffer) for 48 hours at room temperature, washed, and stored in Sorensen's phosphate buffer at 4°C. Stained tibias were arranged in parallel in 1% agarose in a 30‐mL universal tube and mounted in a SkyScan 1172 desktop micro‐CT (Bruker, Kontich, Belgium). The samples were then scanned through 360° using a step of 0.40° between exposures. A voxel resolution of 12.05 μm was obtained in the scans using the following control settings: 54 kV source voltage, 185 μA source current with an exposure time of 885 ms. A 0.5‐mm aluminium filter and two‐frame averaging were used to optimize the scan. After scanning, the data were reconstructed using SkyScan software NRecon v1.6.9.4 (Bruker). The reconstruction thresholding window was optimized to encapsulate the target image. Volumetric analysis was performed using CT Analyzer v1.13.5.1 (Bruker).^24^

### Cell culture and adipocyte differentiation

The 5TGM1‐GFP and JJN‐3 myeloma cell lines were cultured in RPMI media supplemented with 10% fetal bovine serum (FBS), 2 mM L‐glutamine, 1 mM sodium pyruvate, 0.1 mM non‐essential amino acids, and 100 U/mL penicillin‐streptomycin antibiotic, and incubated at 37°C with 5% CO_2_. ST2 cells were seeded in RPMI media (supplemented as above) in 6‐well plates at a density of 8 × 10^5^ per well. After 24 hours, the media was changed to adipogenic media containing 0.5 mM IBMX, 0.25 μM dexamethasone, and 10 μg/mL insulin. Cells were then incubated for 3 days, after which the media was changed to maintenance media containing insulin alone, and cells were then incubated for a further 3 days. Media was then changed to normal RPMI media and incubated for 24 hours before the cells were used. Human bone marrow stromal cells (BMSCs) were grown in DMEM containing 20% FBS. Upon reaching 70% confluence, culture media was changed for adipogenic media (DMEM +10% FBS) containing 0.5 mM IBMX, 1 μM dexamethasone, 100 nM insulin, and 0.2 mM indomethacin. Media was replenished every 3 days for 21 days. Media was then changed to RPMI and cells were incubated for 24 hours before use. Cell differentiation was confirmed using Oil Red O staining according to the manufacturer's instructions. Co‐culture experiments were carried out using transwell inserts (Scientific Laboratory Supplies, Wilford, UK; 353090), which were added to the plates after the differentiation process. Myeloma cells were then plated in the inserts at a density of 1.5 × 10^6^ and left for 24, 48, or 72 hours.

### Lipid uptake studies

Myeloma cells were treated with limiting growth media (RPMI with no glucose or supplements) for 24 hours. Cells were then treated with a 1:1 mix of limiting growth media, limiting growth media and normal media, limiting growth media and BMAd‐derived lipid, or limiting growth media and BMAd 24‐hour conditioned media for a further 24 hours. BMAd‐derived lipid was liberated by scraping the BMAd cells and then centrifuging them at 17,000*g* for 5 minutes. The white lipid layer was then removed from the top of the media, then 150 μL of lipid was added to 600 μL of limiting growth media to make up the BMAd lipid mixture. BMAd conditioned media was media incubated with BMAds for 24 hours. Cells were then seeded in a 96‐well plate for Alamar Blue analysis or in confocal dishes for visualization.

### Oil Red O quantification

Oil Red O stain was eluted using 1 mL of isopropanol for 10 minutes. The absorbance of the elution was measured at a wavelength of 518 nm. Eluted stain from undifferentiated ST2 cells was used as a blank.

### Viability assay

Myeloma cells were seeded in normal RPMI media as described previously at a density of 7.5 × 10^5^/mL in transwell inserts alone or in co‐culture with undifferentiated BMSCs or BM‐like adipocytes derived from ST2 cells. Cells were replated in a 96‐well plate at each time point, then 10% Alamar Blue was added and cells were incubated for 3 hours. Fluorescence intensity was measured using a FLUOstar omega plate reader.

### Migration assay

Boyden chambers (Costar 3422, Corning, Corning, NY, USA) were used according to the manufacturer's instructions. The lower chambers contained either 800 μL normal media, 50% conditioned media taken from undifferentiated ST2 BMSCs (control), or 50% conditioned media from adipocytes derived from ST2 cells. Cells were seeded in the top chamber at a density of 4 × 10^5^ in 200 μL of 1% FBS media. After 24 hours, viability of the cells in the lower chamber was measured using Alamar Blue.

### Patient samples

Serum samples from patients with multiple myeloma, and the respective age‐ and sex‐matched controls (*n* = 20, 15 male, 5 female, aged 62.95 years ±10.7), were obtained through collaboration with MTD and with approval from the Institutional Review Board and Biospecimen Protocol Review Group of the Mayo Clinic College of Medicine. All patients were newly diagnosed myeloma patients (M‐Spike 2.61 g/dL ± 1.99 g/dL, 12 of 15 patients had evidence of bone disease as indicated by osteoporosis and/or lytic lesions or fracture). Bone marrow samples from patients with, or under investigation for, myeloma were obtained with the approval of the Oxford Clinical Research Ethics Committee (09/H0606/5 + 5 project 16/A185).

### QPCR

RNA from cell lines and primary BMSCs was isolated using the RNeasy kit (Qiagen, Valencia, CA, USA). cDNA was generated using Precision Reverse Transcription Premix Kit (Primerdesign, Southampton, UK). Mouse adiponectin was detected by real‐time PCR using Sybr green primers (Life Technologies, Thermo Fisher Scientific). Relative gene expression of adiponectin was normalized to gene expression for *Gapdh* (Life Technologies, Thermo Fisher Scientific). Primer sequences: mAdiponectin F: AGACCTGGCCACTTTCTCCTCATT; mAdiponectin, R: AGAGGAACAGGAGAGCTTGCAAGA; mGAPDH F: TCAACAGCAACTCCCACTCTTC CA; mGAPDH R: ACCCTGTTGCTGTAGCCGTATTCA. QuantiTech Primers for Adipsin, Visfatin, and Resistin were purchased from Qiagen and used according to the manufacturer's instructions.

### Immunoblotting and ELISA

Immunoblotting was used to measure apoptosis and adiponectin expression following a standard Bio‐Rad protocol. The primary antibodies used were 1:1000 rabbit anti‐cleaved PARP (Cell Signaling 5625), 1:1000 rabbit anti‐cleaved caspase‐3 (Cell Signaling 9664), 1:1000 rabbit anti‐adiponectin (Abcam, Cambridge, UK; ab3455), 1:1000 rabbit anti‐phospho‐c‐Jun N‐terminal kinases (p‐JNK; Thr183/Tyr185) (Cell Signaling 9251), 1:1000 rabbit anti‐total JNK (T‐JNK) (Cell Signaling 9252), 1:1000 rabbit anti‐phospho‐p38 mitogen‐activated protein kinases (p‐p38 MAPK; Thr180/Tyr182) (Cell Signaling 4092), 1:1000 rabbit anti‐total p38 MAPK (T‐p38 MAPK) (Cell Signaling 8690), 1:1000 rabbit anti‐phospho‐p44/42 MAPK (phospho‐extracellular signal‐related kinases 1 and 2 [p‐ERK1/2]; Thr202/Tyr204) (Cell Signaling 9101), 1:1000 rabbit anti‐total p44/42 MAPK (T‐ERK1/2) (Cell Signaling 9102), and 1:10,000 mouse anti‐β actin (Sigma A5316). All secondary antibodies were purchased from Cell Signaling. An ELISA was used to measure adiponectin (R&D Systems, Minneapolis, MN, USA; MRP300) and TNF‐α (R&D Systems MHSTA50) according to the manufacturer's instructions.

### mRNA stability assay

ST2‐derived BMAds were treated with recombinant TNF‐α for 24 hours, 10 μg/mL of Actinomycin D was then added, and cells were collected at 0, 1, 2, 4, and 6 hours after treatment. Levels of adiponectin gene expression were measured using qPCR.

### TNF‐α neutralization

Cytokine blocking was achieved using an antibody directed against mouse TNF‐α (R&D Systems MAB4101). Co‐cultures of myeloma cells and mature adipocytes were treated with anti‐TNF‐α at a concentration of 1.5 μg/mL or isotype control. Treated cells were left for 24 hours (5TGM1) or 48 hours (JJN‐3) before conditioned media and RNA were collected.

### Statistical analyses

Statistical significance was determined using a Mann–Whitney *U* test, unpaired *t* test, Pearson correlation or one‐way ANOVA and TUKEY–Kramer post hoc test. Significance was considered for *p* < 0.05. Data sets from in vivo analysis were based on groups of animals. In vitro experiments were performed a minimum of three separate occasions. Data are represented as mean ± SE unless otherwise stated.

## Results

### Bone marrow adiposity is increased in early stage myeloma

**Figure 1:**
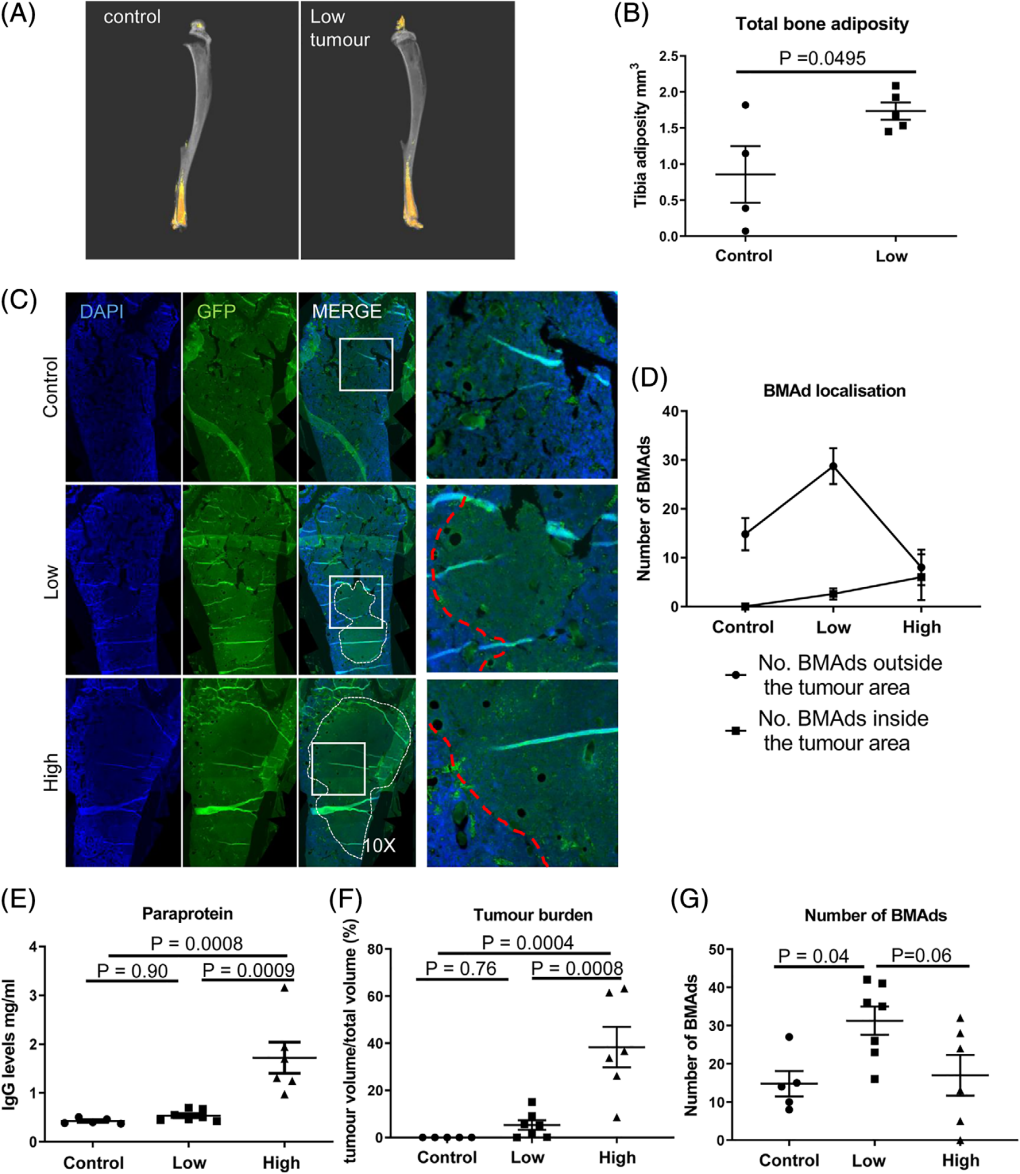
Bone marrow adiposity is increased in early stage multiple myeloma. (*A*) Osmium tetroxide–stained tibia analyzed in a model of low tumor burden, <10% myeloma cells in bone marrow. (*B*) Total bone adiposity was measured using CT Analyzer v1.13.5.1 (Bruker, Kontich, Belgium) software.^24^ (*C*) Immunofluorescence imaging using anti‐GFP was used to visualize the tumor area and BMAd localization in the femur (white dotted line highlights area of tumor, red dotted line denotes tumor‐bone interface). (*D*) Quantitation of BMAd number, inside and outside of the tumor area. (*E*) Paraprotein levels measured by ELISA. (*F*) Tumor burden was calculated using osteometric software. (*G*) The number of BMAds was counted using osteometric software. Data represent the mean ±SE.

To investigate the effect of myeloma cells on BMAd localization and number, the well‐established Radl model of myeloma was used.^25^, ^26^ This model successfully recapitulates human disease with 5TGM1 MM cells homing to bone, where they become established and cause bone lesions.^27^, ^28^ First, we analyzed KaLwRij mice with low tumor burden using osmium tetroxide staining of their tibiae (Fig. 1 *A*). {FIG1} These mice had significantly higher bone adiposity compared with controls (Fig. 1 *B*), and this increase in adiposity was primarily confined to the distal tibia, suggesting that the increase is within the constitutive BMAd population.^29^ We did not observe any changes in the adipocytes that reside in the proximal tibia, although this may reflect the sensitivity of osmium‐tetroxide–based detection. To investigate this further, mice were culled 16 and 23 days post inoculation to mimic low and high tumor burden, reflecting early and late‐stage disease. Immunofluorescence of the femurs confirmed the presence of GFP‐positive tumor cells (denoted by white line), as well as identifying BMAds morphologically using adipocyte marker, perilipin (Fig. 1 *C*, Supplemental Fig. S1). We observed that the BMAds present in the 23‐day mice were predominantly located along the tumor‐bone interface (Fig. 1 *C*, red dotted line), with a change in the ratio of BMAds outside the tumor area/within the tumor area from 9:1 in the low tumor burden mice to 1:1 in the high tumor burden mice (Fig. 1 *D*). Similar localization of BMAds along the tumor‐bone interface was observed in a JJN‐3 model of myeloma (Supplemental Fig. S2). Paraprotein levels and tumor burden were significantly higher in the mice with high tumor burden compared with low (Fig. 1 *E*, *F*), indicating significant disease progression from less than 10% plasma cells within the bone marrow to greater than 30% marrow infiltration. The number of BMAds present in low tumor mice was significantly higher in plasma cell–inoculated mice compared with control. However, this increase was not observed in the high tumor mice (Fig. 1 *G*).

### BMAds support myeloma growth by a lipid‐independent mechanism

**Figure 2:**
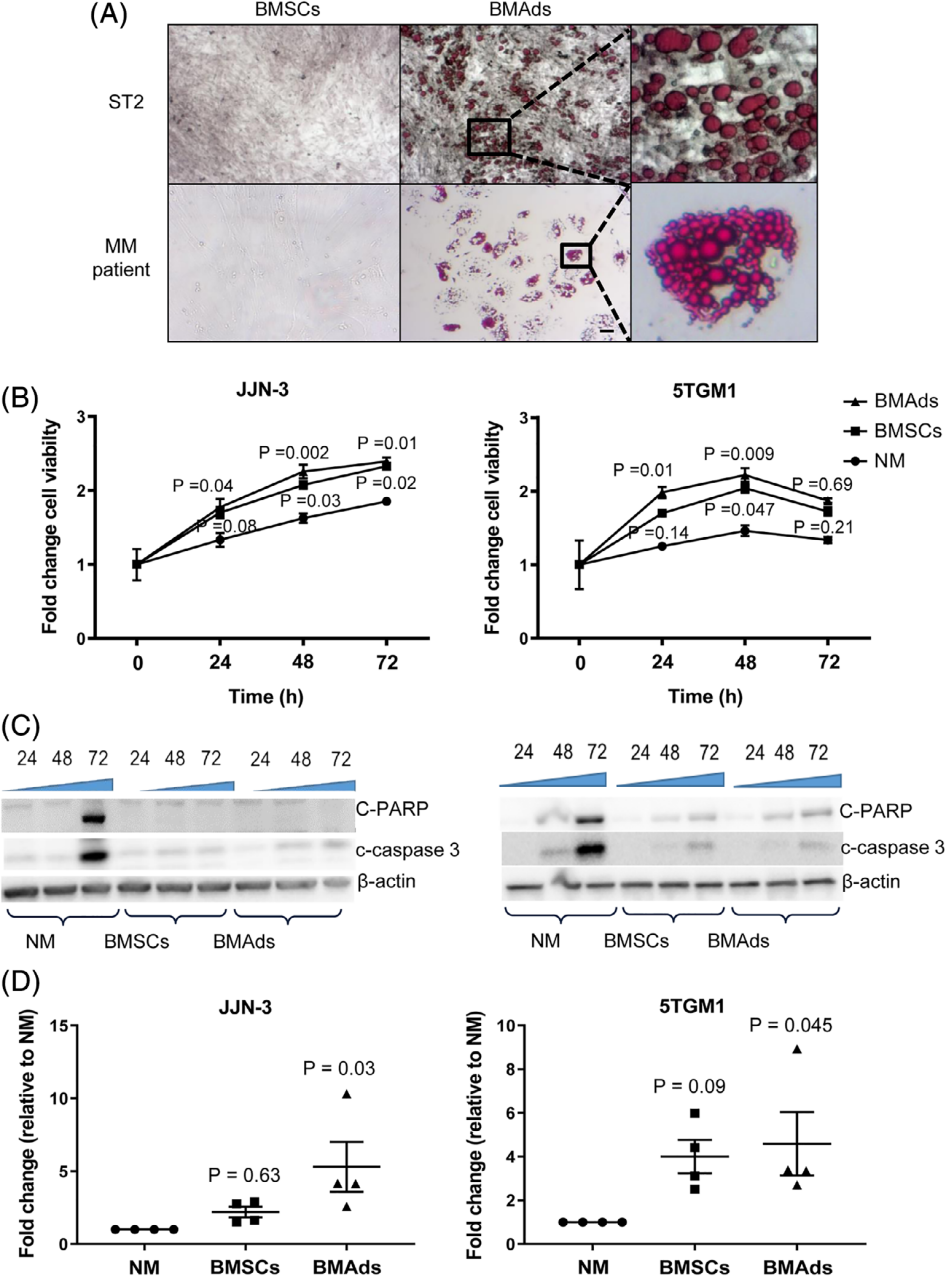
BMAds support myeloma growth and survival. (*A*) ST2 cell line BMSCs and patient‐derived BMSCs treated with an adipogenic cocktail. Scale bar = 200 μm. (*B*) Viability of myeloma cell lines was measured using Alamar blue after co‐culture with either normal media alone (NM), BMSCs, or BMAds. Results are expressed as fold change relative to control cultures. Cells were cultured in normal RPMI media. Data represent the mean ±SE of three independent experiments. Statistical significance was calculated compared with NM. (*C*) The expression of apoptotic markers was assessed by immunoblotting. (*D*) Migration was assessed using Boyden chambers. Cell number in the lower chamber was measured using Alamar blue. Data represent the mean ±SE of four independent experiments. Statistical significance was calculated compared with NM control.

Our discovery that myeloma cells promote an increase in the number of BMAds at early stages of disease in vivo, combined with previous studies suggesting that BMAds are supportive of myeloma growth,^9^, ^11^ led us to consider how BMAds can confer a survival advantage to myeloma cells. Our studies confirm the myeloma‐supportive effects of BMAds, with a significant increase in myeloma cell viability and decrease in apoptosis when myeloma cells were cultured with either BMAds or BMSCs and an increase in migration toward media conditioned from BMAds (Fig. 2 *A*–*D*). {FIG2}

**Figure 3:**
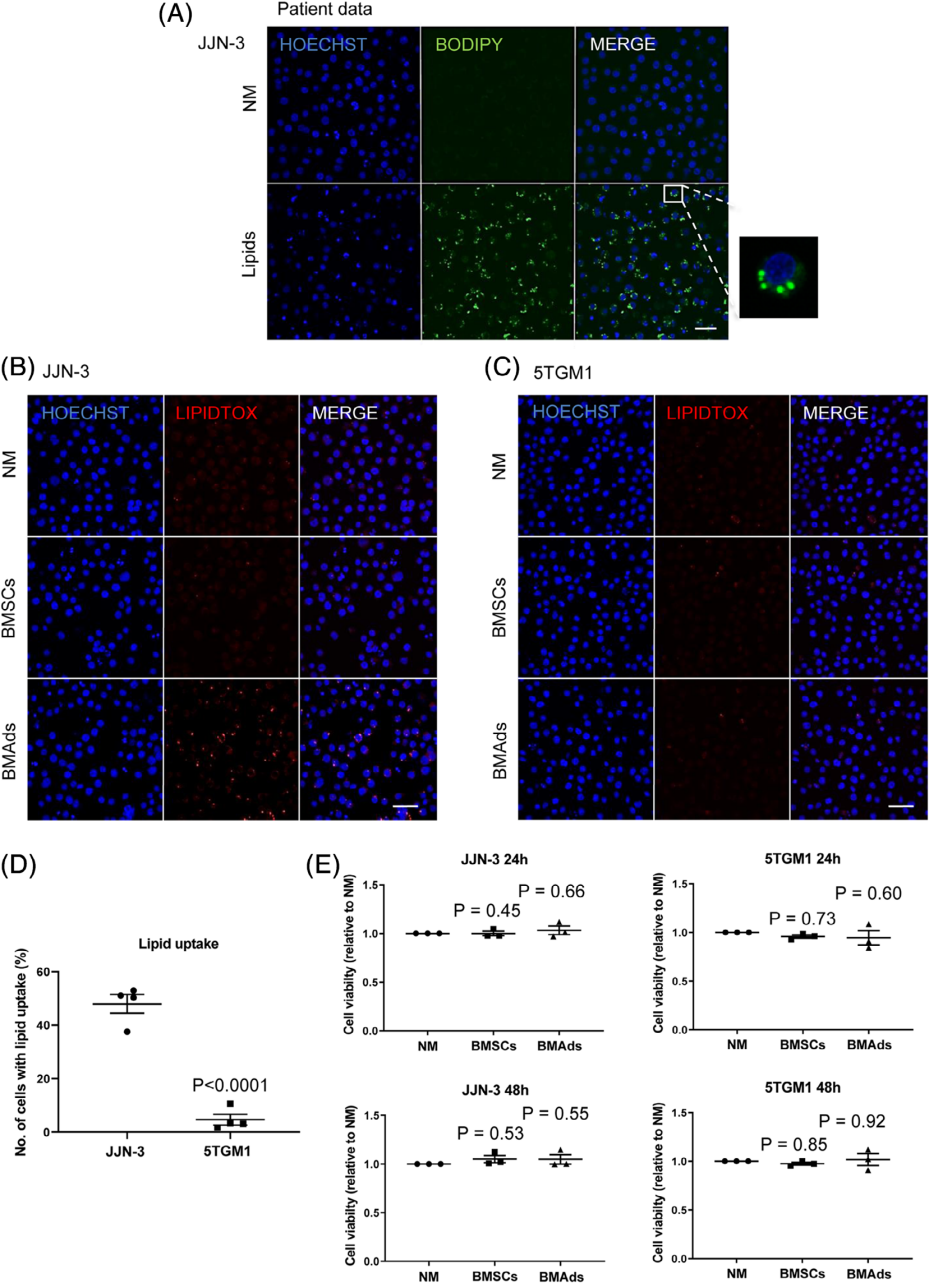
BMAds support myeloma cell growth by a lipid‐independent mechanism. (*A*) BODIPY 493/503 staining of myeloma patient‐derived lipid droplets (green) in JJN‐3 cells. Scale bar = 50 μm. (*B*) Lipidtox neutral red staining of ST2‐derived lipid droplets in JJN‐3 cells. Scale bar = 50 μm. (*C*) Lipidtox neutral red staining of ST2‐derived lipid droplets in 5TGM1‐GFP cells. Scale bar = 50 μm. All microscopy images show a representative image from four independent experiments. (*D*) The percentage of cells exhibiting lipid droplets was calculated using ImageJ software. Data points represent the mean ±SE of four independent experiments. (*E*) Viability was measured using Alamar blue in JJN‐3 and 5TGM1 cells after incubation with normal media (NM), NM with supernatant from scraped ST2 BMSCs, or NM with ST2 BMAd‐derived lipid droplets. Data points represent the mean ±SE of three independent experiments. Statistical significance was calculated compared with NM control.

**Figure 4:**
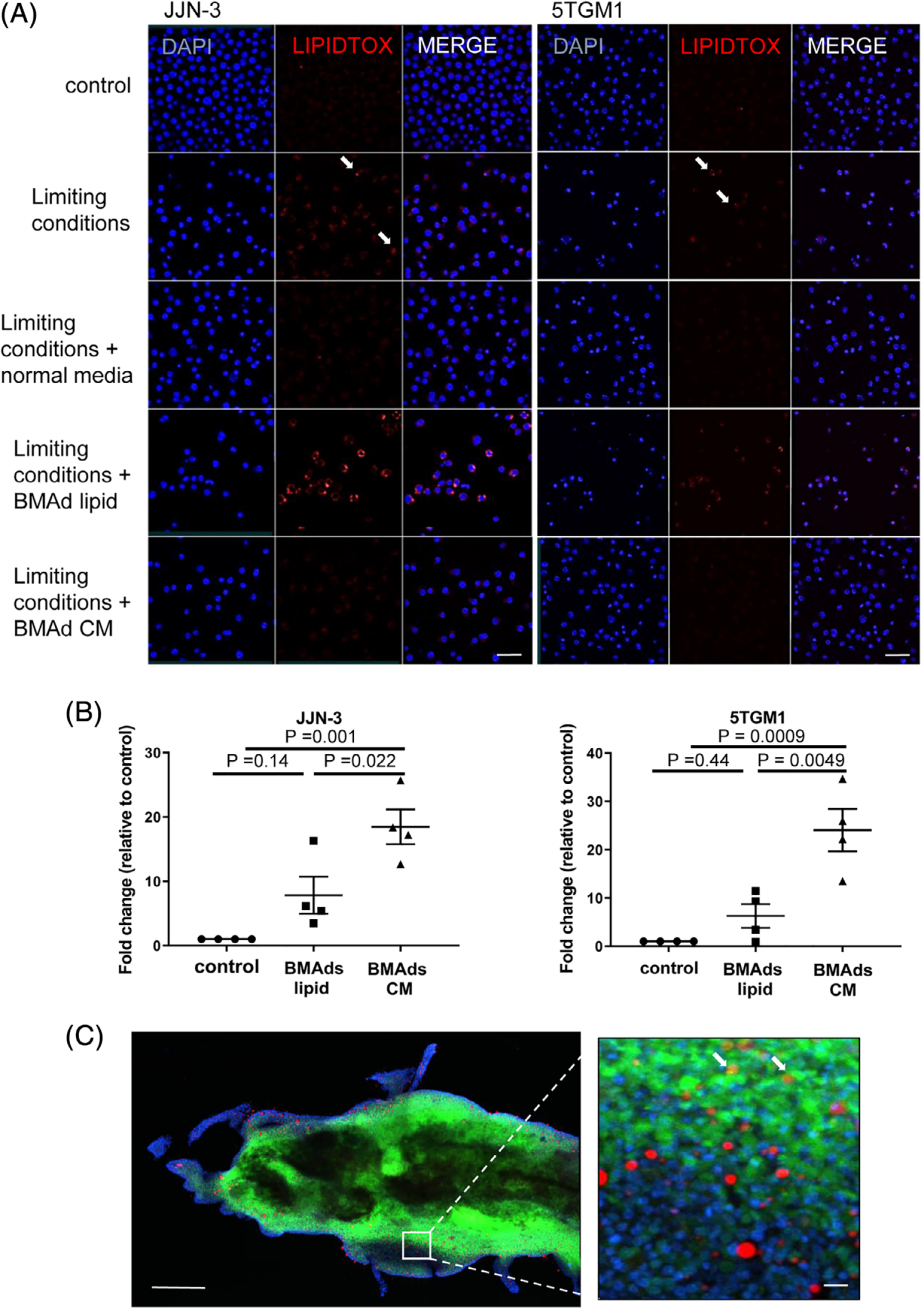
Growth‐limiting conditions induce de novo lipogenesis in myeloma cells. (*A*) Lipidtox neutral red staining of ST2‐derived lipid droplets in JJN‐3 and 5TGM1 cells cultured in limiting conditions and treated with liberated lipids or conditioned media from BMAds. Scale bar = 50 μm. All microscopy images show a representative image from three independent experiments. (*B*) Viability was measured using Alamar blue. Results are expressed as fold change relative to control cultures. Cells were cultured under limiting conditions for 24 hours followed by the addition of BMAd‐derived lipid or BMAd conditioned media for a further 24 hours. Data represent the mean ±SE of four independent experiments. Statistical significance was calculated compared with limiting media control. (*C*) Bone marrow plug taken from a 6‐month‐old mouse 23 days post inoculation with 5TGM1 myeloma cells. Immunofluorescence of 5TGM1‐GFP (green) cells and Lipidtox (red) to visualize adipocytes/lipid. Scale bars = 200 and 50 μm, respectively.

BMAds are a major source of lipid, and prostate cancer cells are known to uptake adipocyte‐derived lipids to promote cell viability.^5^ This led us to investigate whether myeloma cells behave in a similar manner. Visualization of lipid droplets demonstrated that JJN‐3 myeloma cells were able to uptake lipids isolated from the bone marrow of patients with myeloma (Fig. 3 *A*). {FIG3} However, when JJN‐3 or 5TGM1 myeloma cells were incubated with liberated lipid droplets from BMSCs or BMAds, the amount of lipid uptake was significantly different between the myeloma cells, with limited lipid uptake detected in 5TGM1 myeloma cells (Fig. 3 *B*–*D*). Next, we investigated whether lipid uptake had a positive effect on myeloma cell viability. There was no difference in viability between JJN‐3 or 5TGM1 myeloma cells that had been incubated with or without BMAd‐derived lipid (Fig. 3 *E*). Cells were then placed under metabolic stress to establish whether by inducing a stress response, they may utilize lipid for survival. Myeloma cells were grown in limiting condition media (RPMI with no glucose, supplements, or FBS) for 24 hours and then incubated with liberated lipid or conditioned media from BMAds. Interestingly, the culture of myeloma cells in limiting conditions resulted in the appearance of lipidtox‐positive cells, indicative of de novo lipogenesis (Fig. 4 *A*). {FIG4} A trend toward increased viability was observed when myeloma cells were cultured in limiting conditions and then exposed to BMAd‐derived lipid. However, conditioned media taken from BMAds was found to significantly increase viability compared with both the control and the BMAd‐derived lipid (Fig. 4 *B*). To determine whether lipid is present within myeloma cells in vivo, we used a technique to visualize the bone marrow by extracting fresh marrow with a needle and fixing and staining it immediately.^22^ Using this technique, we were only able to detect the presence of lipid in a very small number of 5TGM1‐GFP cells present in the bone marrow in vivo (Fig. 4 *C* and Supplemental Fig. S3). Furthermore, it is not possible to conclude whether these lipid droplets were from neighboring BMAds or a product of de novo lipogenesis.

### Myeloma cells downregulate adiponectin in BMAds

**Figure 5:**
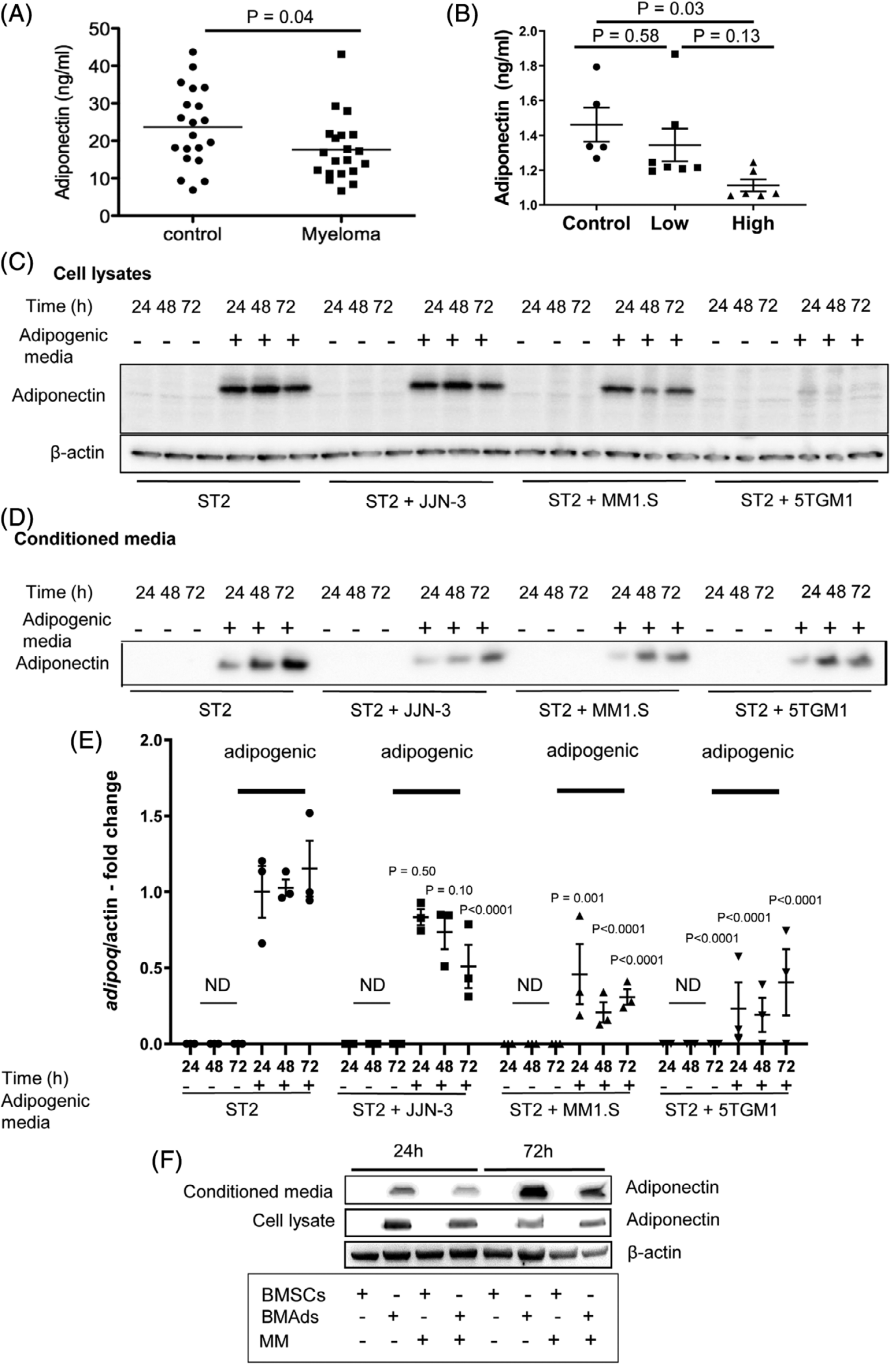
Myeloma cells downregulate adiponectin in BMAds. (*A*) Adiponectin expression in human serum. (*B*) Bone marrow plasma concentrations of adiponectin in an experimental cohort of KaLwRij mice. (*C*) Adiponectin expression in ST2 cells co‐cultured with myeloma cell lines for 24, 48, and 72 hours was assessed by immunoblotting. (*D*) Secreted adiponectin levels in conditioned media from ST2 cells co‐cultured with myeloma cell lines for 24, 48, and 72 hours were assessed by immunoblotting. Equal volumes of conditioned media were loaded into each lane. (*E*) *Adipoq* expression in ST2 cells co‐cultured with myeloma cells for 24, 48, and 72 hours was assessed using RT‐PCR (ND = not detectable). Data points represent the mean ±SE of three independent experiments. Statistical significance was calculated compared to ST2 alone control (no adipogenic media). (*F*) Adiponectin expression in ST2 cells co‐cultured with primary human myeloma cells for 24 and 72 hours was assessed by immunoblotting.

Our studies reveal a myeloma‐promoting effect of BMAds that does not appear to be related to lipid uptake. To reconcile our findings that BMAds are increased and protect myeloma cells despite the known tumor‐suppressive effects of adiponectin,^16^ we investigated whether myeloma cells downregulate adiponectin in BMAds. Quantitation of circulating adiponectin revealed a significant reduction in adiponectin in patients with multiple myeloma and in myeloma‐bearing mice (Fig. 5 *A*, *B*). {FIG5} Co‐cultures of myeloma cells with BMSCs or BMAds demonstrated that adiponectin was significantly downregulated in the ST2‐derived BMAds that had been co‐cultured with myeloma cells compared with BMAds cultured with media alone. This was observed in both cell lysates and conditioned media at the protein level (Fig. 5 *C*, *D*) as well as at the RNA level (Fig. 5 *E*). To further confirm the downregulation of adiponectin in BMAds, primary myeloma cells were co‐cultured with ST2‐derived BMAds (Fig. 5 *F*), again showing a decrease in adiponectin levels after 24 hours of co‐culture. This reduction was not observed after 72 hours in the cell lysates; however, this may be a reflection of low primary cell viability after 72 hours of culture. Similar experiments were performed using 3T3‐L1 adipocytes. Adiponectin secretion was lower in 3T3‐L1 adipocytes than in ST2‐derived BMAds, confirming that the former are more like white adipose tissue (WAT) (Supplemental Fig. S4*A*). However, unlike in ST2‐derived BMAds, myeloma cells did not further decrease adiponectin expression in 3T3‐L1 adipocytes, suggesting that the ability of myeloma cells to downregulate adiponectin is specific to BMAds (Supplemental Fig. S4*B*).

### Myeloma cells downregulate adiponectin in BMAds via TNF‐α

**Figure 6:**
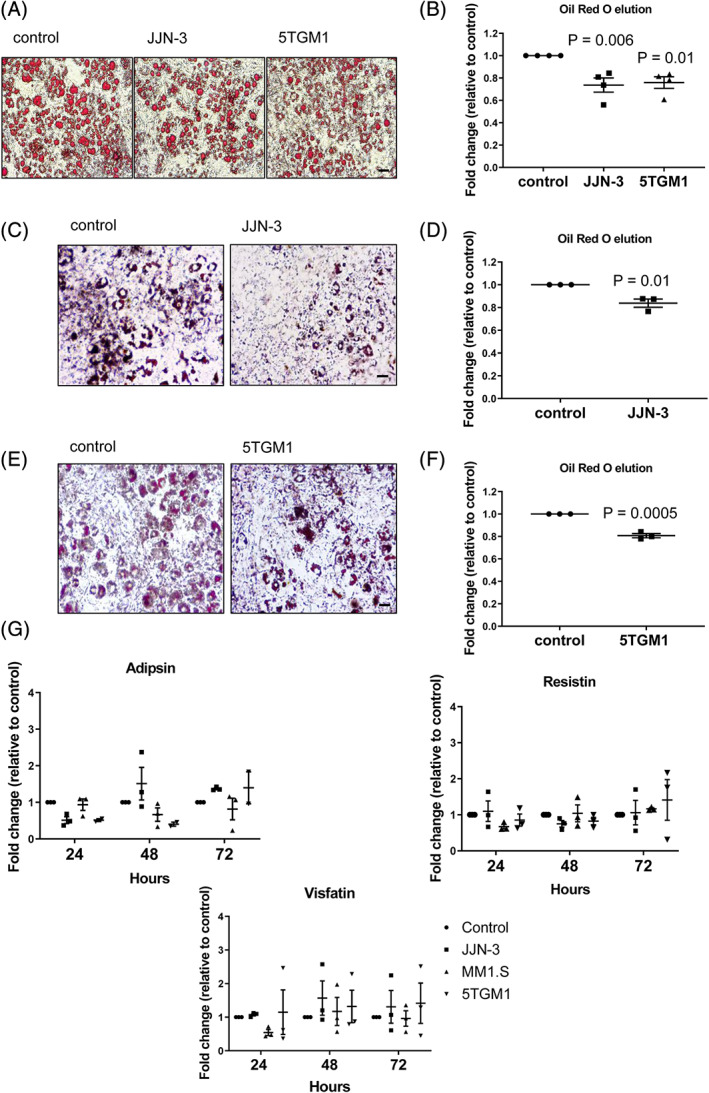
Myeloma cells reduce BMAd size and number without causing a generalized loss in adipokine secretion. (*A*) Oil Red O–stained BMAds after 72 hours of direct co‐culture with myeloma cells. Scale bar = 50 μm. (*B*) Eluted Oil Red O stain from BMAds after direct co‐culture with JJN‐3 or 5TGM1 cells. (*C*) BMAds after 72 hours of transwell co‐culture with JJN‐3 cells. Scale bar = 50 μm. (*D*) Eluted Oil Red O stain from BMAds after transwell co‐culture with JJN‐3 cells. (*E*) BMAds after 72 hours of transwell co‐culture with 5TGM1 cells. Scale bar = 50 μM. (*F*) Eluted Oil Red O stain from BMAds after transwell co‐culture with 5TGM1 cells. Data points represent the mean ±SE of three independent experiments. Statistical significance was calculated compared with ST2 alone control. (*G*) *CFD/*Adispsin, *ADSF/*Resistin, and *Nampt/*Visfatin expression was assessed by RT‐PCR. Data points represent the mean ±SE of three independent experiments, *p* > 0.05. Exact *p* values are shown in Supplemental Table S1.

**Figure 7:**
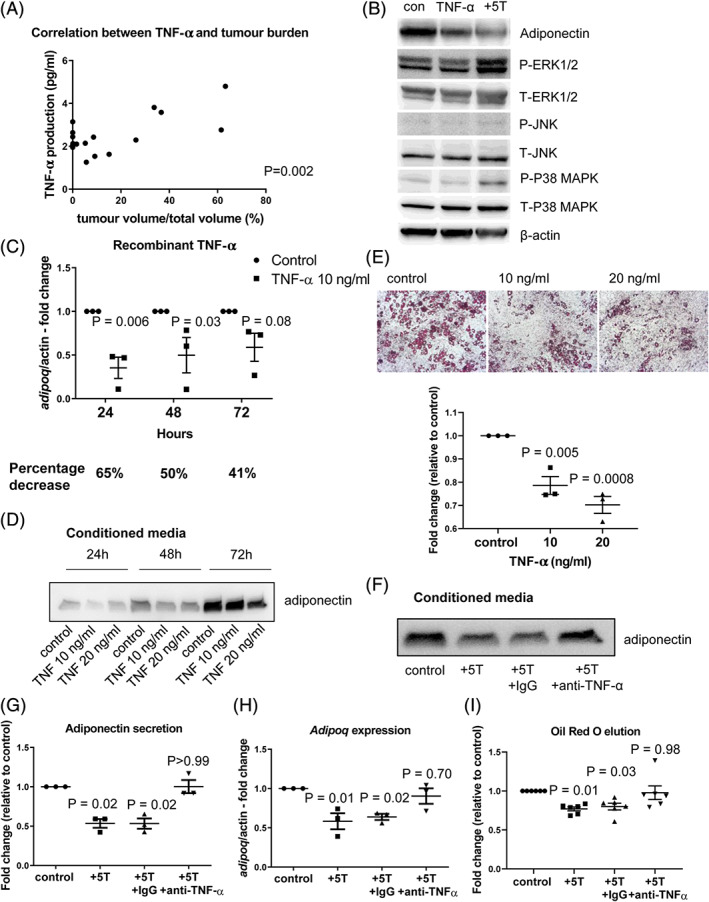
TNF‐α downregulates adiponectin in BMAds. (*A*) Correlation between bone marrow plasma–derived TNF‐α and tumor burden from 5TGM1 myeloma‐bearing mice. (*B*) Myeloma cells activate ERK1/2 signaling in BMAds. Protein levels of Adiponectin, P‐ERK1/2, T‐ERK1/2, P‐JNK, T‐JNK, P‐P38 MAPK, and T‐P38 MAPK were assessed by immunoblotting in ST2‐derived BMAds after 24‐hour treatment with 10 ng/mL recombinant TNF‐α or co‐culture with 5TGM1 cells. (*C*) *Adipoq* expression in ST2‐derived BMAds after treatment with 10 ng/mL mouse recombinant TNF‐α for 24, 48, and 72 hours was measured by RT‐PCR. Percentage decrease was calculated compared with time point control. (*D*) Protein levels of adiponectin in conditioned media taken from ST2‐derived BMAds after 10 or 20 ng/mL mouse recombinant TNF‐α treatment for 24, 48, or 72 hours were assessed by immunoblotting. Equal volumes of conditioned media were loaded into each lane. (*E*) BMAds were treated with 10 or 20 ng/mL mouse recombinant TNF‐α for 72 hours. Cells were fixed and stained with Oil Red O stain. Scale bar = 50 μm. Stain was eluted and absorbance measured. (*F*) BMAds were co‐cultured with myeloma cells (5TGM1) in the presence or absence of an anti‐TNF‐α neutralizing antibody. Secreted adiponectin level in the conditioned media was measured. Equal volumes of conditioned media was loaded into each lane. (*G*) Densitometry using ImageJ software was used to quantify the level of secreted adiponectin shown in *F*. (*H*) BMAds were cultured with myeloma cells (5TGM1) in the presence or absence of an anti‐TNF‐α neutralizing antibody and *Adipoq* expression was assessed by RT‐PCR. Data points represent the mean (±SE) of three independent experiments. Statistical significance was calculated compared with control. (*I*) BMAds were co‐cultured with myeloma cells (5TGM1) in the presence or absence of an anti‐TNF‐α neutralizing antibody for 72 hours. BMAds were fixed and stained with Oil Red O. Stain was eluted and absorbance measured. Data points represent the mean ±SE of six independent experiments. Statistical significance was calculated compared with control.

Our data demonstrate that myeloma cells downregulate adiponectin in BMAds. However, the mechanism behind this downregulation remains unknown. Co‐culture of myeloma cells with BMAds, either in direct contact or separated by a transwell membrane, was found to have a modest effect to reduce BMAd number and size (Fig. 6 *A*–*F*). {FIG6} Investigation of additional adipokines did not reveal a generalized loss in adipokine secretion, with no significant difference in expression of resistin, visfatin, or adipsin (Fig. 6 *G*). This suggests a specific mechanism underlying the downregulation of adiponectin in BMAds by myeloma cells. Myeloma cells secrete a number of cytokines known to drive disease progression, one of which is TNF‐α.^30^, ^31^ Measurement of TNF‐α in the bone marrow plasma of myeloma‐bearing mice demonstrated a significant correlation with tumor burden, confirming an association with disease progression (Fig. 7 *A*). {FIG7} Previously TNF‐α has been implicated as a potential regulator of adiponectin expression in WAT but not in the context of cancer.^32^, ^33^, ^34^ Transwell co‐culture of myeloma cells with BMAds was found to increase activation of JNK, p38MAPK, and ERK1/2, pathways which have previously been implicated in TNF‐α‐mediated suppression of adiponectin in WAT^35^ (Fig. 7 *B*). TNF‐α treatment alone did not induce these changes in BMAds, suggesting that WAT may be more sensitive to TNF‐α alone. To determine whether TNF‐α could regulate adiponectin in BMAds, we treated ST2‐derived BMAds with mouse recombinant TNF‐α. After 24 and 48 hours, there was a 65% and 50% respective decrease in *Adipoq* expression (Fig.7 *C*) and in secreted adiponectin (Fig. 7 *D*). A smaller reduction in *Adipoq* expression (41%) was observed after 72 hours, suggesting a level of recovery from the initial TNF‐α exposure. TNF‐α treatment also resulted in a significant difference in *Adipoq* mRNA stability after a 2‐hour treatment with the transcriptional inhibitor actinomycin D but no overall change in mRNA half‐life (Supplemental Fig. S5). The addition of recombinant TNF‐α to mature BMAd cultures for 72 hours also caused a reduction in Oil Red O staining, indicating a decrease in BMAd number in a similar manner to myeloma/BMAd co‐culture (Fig. 7 *E*). To determine whether the downregulation of adiponectin was due to TNF‐α, a TNF‐α blocking antibody was used. Adiponectin secretion (Fig. 7 *F*–*G*) and *Adipoq* expression (Fig. 7 *H* and Supplemental Fig. S6*A*) were decreased in co‐cultures containing either 5TGM1 or JJN‐3 myeloma cells alone or in the presence of an isotype control antibody. In contrast, addition of a neutralizing antibody to TNF‐α to myeloma/BMAd co‐cultures had no effect on viability (Supplemental Fig. S6*B*) but blocked the suppression of adiponectin and prevented the reduction in BMAd number (Fig. 7 *I* and Supplemental Fig. S6*C*).

## Discussion

There is growing evidence that BMAds play an important role in a number of different cancers that metastasize to (breast and prostate) or develop within the bone, such as myeloma. Our study advances these previous findings by providing a mechanism by which myeloma cells interact with BMAds to circumvent the tumor‐suppressive effect of adiponectin.

Using our murine myeloma model, we have shown that in early stage disease (defined clinically as less than 10% myeloma cells present within the bone marrow) there is a significant increase in marrow adiposity. This is supported by previous studies that revealed that bone marrow from patients with myeloma contained increased pre‐adipocytes and significantly larger mature adipocytes than bone marrow obtained from healthy volunteers and that adipogenic gene expression is elevated in mesenchymal stem cells from myeloma‐bearing mice.^10^, ^12^ Our data show an increase in overall adiposity volume, which could be due to either increased BMAd size or number or both. This increase may be in response to the initial influx of cancer cells, as it is not observed in late disease. Although this finding is to be expected, as in end‐stage disease, the myeloma cells have substantially increased in number and both modulate bone cell numbers and behavior and also physically crowd out other cells within the bone marrow microenvironment. Therefore, the observed change in ratio from BMAds localized outside the tumor area to BMAds inside the tumor area in the high‐tumor‐bearing mice may be a simple reflection of increased tumor burden. However, interestingly, the remaining BMAds were shown to be lining the tumor‐bone interface, suggesting that BMAd localization is affected by the infiltrating tumor cells. Whether these BMAds are recruited by the tumor cells, whether BMAds within the tumor area are destroyed by the infiltrating tumor cells, leaving only the cells surrounding the tumor, or whether the remaining BMAds shrink in size but persist in the marrow remains unclear. Our in vitro studies demonstrate a small reduction in BMAd number and size after culture with myeloma cells, supporting our in vivo findings in late‐stage myeloma. The initial increase in adiposity that we observed suggests that BMAds have a positive impact on disease progression. An intriguing finding was that the increase in adiposity appeared most prominently in the distal section of bone. BMAds are proposed to exist in two broad subtypes: regulated and constitutive. Regulated BMAds are found in the more metabolically active regions of the bone and are thought to be responsible for responding to environmental cues such as changes in diet or exercise. Constitutive BMAds develop early in life and are rarely depleted and are thought to be less responsive than regulated BMAds.^3^ However, in our study, we identified an increase in marrow adiposity in the distal tibia, the site at which constitutive BMAds reside. One possibility is that myeloma does impact the regulated BMAds but that in our model these cells remain too diffuse to be detectable by osmium tetroxide staining. Indeed, KaLwRij mice are a sister strain of the B6 mouse, which have low levels of regulated fat until later in life.^29^ At younger ages, as in our mice, regulated BMAds are dispersed among hematopoietic cells, whereas constitutive BMAds are clustered together resembling the tightly packed structure of adipocytes in WAT. As such, our findings in the distal tibia might reflect the sensitivity of the osmium staining for detection of clusters of BMAds as opposed to single cells. If so, in our model, changes in regulated BMAds within the metaphyseal region may be below the level of detection using this approach. It is also possible that early changes occur in regulated BMAds residing close to the constitutive region but that these changes are too subtle to detect. Alternatively, it may be that myeloma cells modify only the constitutive BMAds. It must also be acknowledged that a limitation of the study is that histological analysis was performed on femurs and osmium tetraoxide on tibiae, and it is of interest to determine whether changes in constitutive BMAds are also observed in femoral bone. Formally addressing these questions in future studies is warranted.

Our findings also provide evidence to support the growing belief that BMAd interactions are advantageous for myeloma cell viability and evasion of apoptosis and that adipogenic factors promote myeloma cell migration.^9^, ^10^, ^11^ Interestingly, we also observed that BMSCs offer a significant level of support in promoting viability and evading apoptosis, which raises the question as to the similarities between these two cell types and the factors they secrete. In contrast, BMAds significantly increased the migration rates of myeloma cells compared with BMSCs. This is in line with other studies in different tumor types that have a propensity to metastasize to bone; one study showed that breast cancer cells have increased migration toward human bone tissue‐conditioned medium^7^ and another that prostate cancer cells interact with BMAds, resulting in the promotion of growth and invasiveness driven by BMAd‐derived lipids from nearby BMAds.^5^ These observations raise the questions as to whether myeloma cells are attracted to factors secreted by BMAds or whether myeloma cells need to utilize the BMAd's lipid storage in a manner similar to prostate tumor cells, or whether both elements are important. We have shown that conditioned media rich in BMAd‐derived factors promotes myeloma cell growth and migration. Furthermore, we have also shown that myeloma cells are capable of BMAd‐derived lipid uptake in vitro; however, striking differences were found between myeloma cell lines. Interestingly we did not observe a difference in viability when liberated BMAd‐derived lipid was added to normal culture media. However, we did observe a trend toward elevated viability when myeloma cells were cultured in growth‐limiting conditions and then liberated lipid was added, suggesting that lipids may be utilized in times of stress. In addition, growth‐limiting conditions alone induced de novo lipogenesis in myeloma cells, suggesting a selective dependence upon lipid as an energy source that warrants further investigation. In vivo, the presence of lipid was detectable in a small proportion of myeloma cells in the bone marrow of myeloma‐bearing mice. Although it is not possible to distinguish between lipid uptake and de novo lipogenesis in vivo, the small proportion of myeloma cells with detectable lipid may suggest a limited role for lipid in myeloma growth in the bone marrow. Notably, although there is a marked difference in lipid uptake between 5TGM1 and JJN‐3 myeloma cells in vitro, the in vivo preclinical models are strikingly similar with respect to tumor burden and osteolytic bone disease. Furthermore, we show that localization of BMAds along the tumor‐bone interface is comparable between models. Taken together, our data suggest a limited role for lipid uptake in disease pathogenesis. Future studies are needed to elucidate whether lipids supply valuable energy for cell motility and/or invasiveness or whether they may play a direct role in conferring a level of drug resistance.

Most research in the adipocyte‐cancer field implicates the adipocyte as a cell that promotes tumor growth. However, adipocytes do not secrete only tumor‐promoting factors. One adipokine known to have potent antitumor effects is adiponectin, the most highly expressed adipokine in the WAT. However, low adiponectin levels are associated with a variety of disorders including coronary heart disease,^36^ type 2 diabetes,^37^, ^38^ obesity, and cancer.^39^ Interestingly, a number of these conditions have also been associated with high levels of TNF‐α.^40^, ^41^, ^42^ Patients with myeloma were shown to have significantly higher levels of TNF‐α compared with healthy volunteers, with levels rising in association with disease progression.^43^ Our murine data support these findings, showing that levels of TNF‐α also correlate with tumor burden and that mice inoculated with myeloma cells also have significantly lower adiponectin levels. These findings extend our previous studies, where we found that adiponectin was reduced in myeloma‐permissive mice.^16^ However, the mechanism(s) that links these two proteins in the development of myeloma is unknown. TNF‐α is known to regulate adiponectin production in WAT. We now show that TNF‐α is able to downregulate adiponectin in ST2‐derived BMAds, likely dependent on both transcriptional regulation and loss of mRNA stability. More importantly, we show that myeloma cells are able to manipulate their environment by downregulating adiponectin in these BMAds, at least in part via the secretion of TNF‐α. Moreover, the addition of recombinant TNF‐α also caused a change in BMAd number in a similar manner to myeloma/BMAds co‐cultures. This reduction in adiposity may also contribute to lower levels of adiponectin, especially in late‐stage disease as the myeloma cells start to overpopulate the marrow and directly interact with the majority of remaining BMAds. The addition of a TNF‐α‐neutralizing antibody restored both adiponectin expression levels and BMAd number; however, interestingly, viability was not altered by blockade of TNF‐α, suggesting additional mechanisms may contribute to the effect of BMAds on myeloma cell viability. Elevated levels of TNF‐α have long been associated with myeloma, and our results provide new insights into the mechanisms by which this key inflammatory cytokine promotes myeloma disease progression.

Collectively, our studies provide new insights into the BMAd‐myeloma cell interaction in vivo, revealing a new mechanism by which myeloma cells alter their microenvironment to support their growth. BMAd‐derived factors are utilized for growth and survival and in order to fully take advantage of this relationship, myeloma cells have developed a valuable mechanism to downregulate adiponectin to thereby evade its antitumor effects. Future studies are needed to further investigate and understand the contribution of BMAd‐derived lipids and whether they play a significant role in myeloma disease progression. Once thought of as an inert space‐filling cell, BMAds are slowly emerging as fundamental to disease progression. As society battles against the burgeoning obesity epidemic, efforts to more fully understand the implications of increased bone marrow adiposity for tumor growth and survival within bone will become increasingly important.

## Disclosures

All authors state that they have no conflicts of interest.

## Supporting information


**Supplemental Fig. S1**.
Supplemental Fig. S2.
Supplemental Fig. S3.
Supplemental Fig. S4.
Supplemental Fig. S5.
Supplemental Fig. S6.
**Supplemental Table S1.** The *p* Values Associated With Fig. 6 *G*
