# Supplementary material for: Myeloma Cells Down‐Regulate Adiponectin in Bone Marrow Adipocytes Via TNF‐Alpha
Source: J Bone Miner Res. 2020 Jan 16;35(5):942–55. doi: 10.1002/jbmr.3951 (PMC9328417; doi:10.1002/jbmr.3951)
Supplement: Supplementary file 1 — Supplemental Fig. S1. Supplemental Fig. S2. Supplemental Fig. S3. Supplemental Fig. S4. Supplemental Fig. S5. Supplemental Fig. S6. Supplemental Table S1. The p Values Associated With Fig. 6 G [file JBMR-35-942-s001.pdf]

Supplemental Data

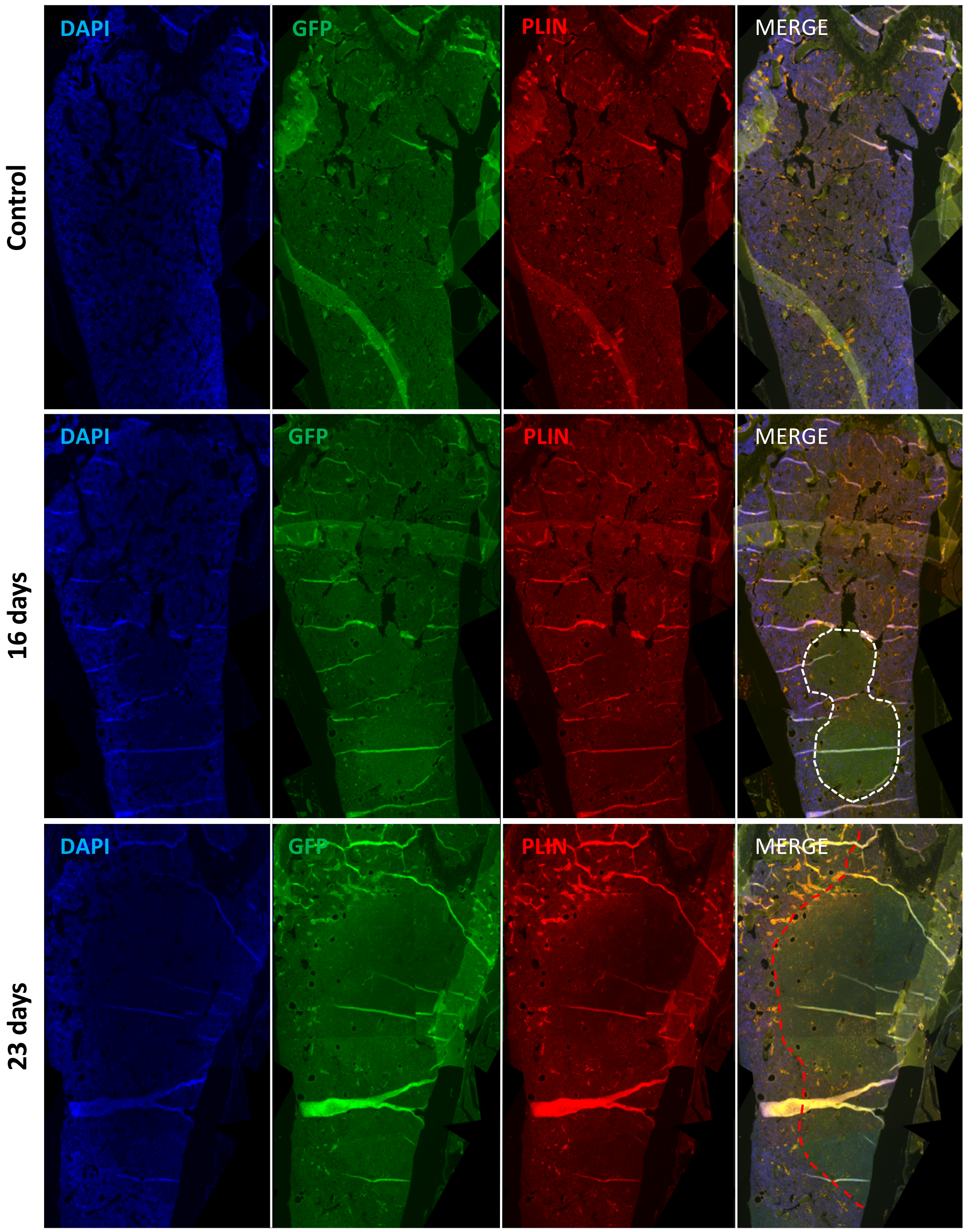

Supplemental Figure 1. BMAds predominantly locate along the tumour-bone interface. Formalin fixed sections stained with anti-GFP and Perilipin to visualise BMAd location in the femur (white circle highlights tumour area, red dotted line denotes bone-tumour interface).

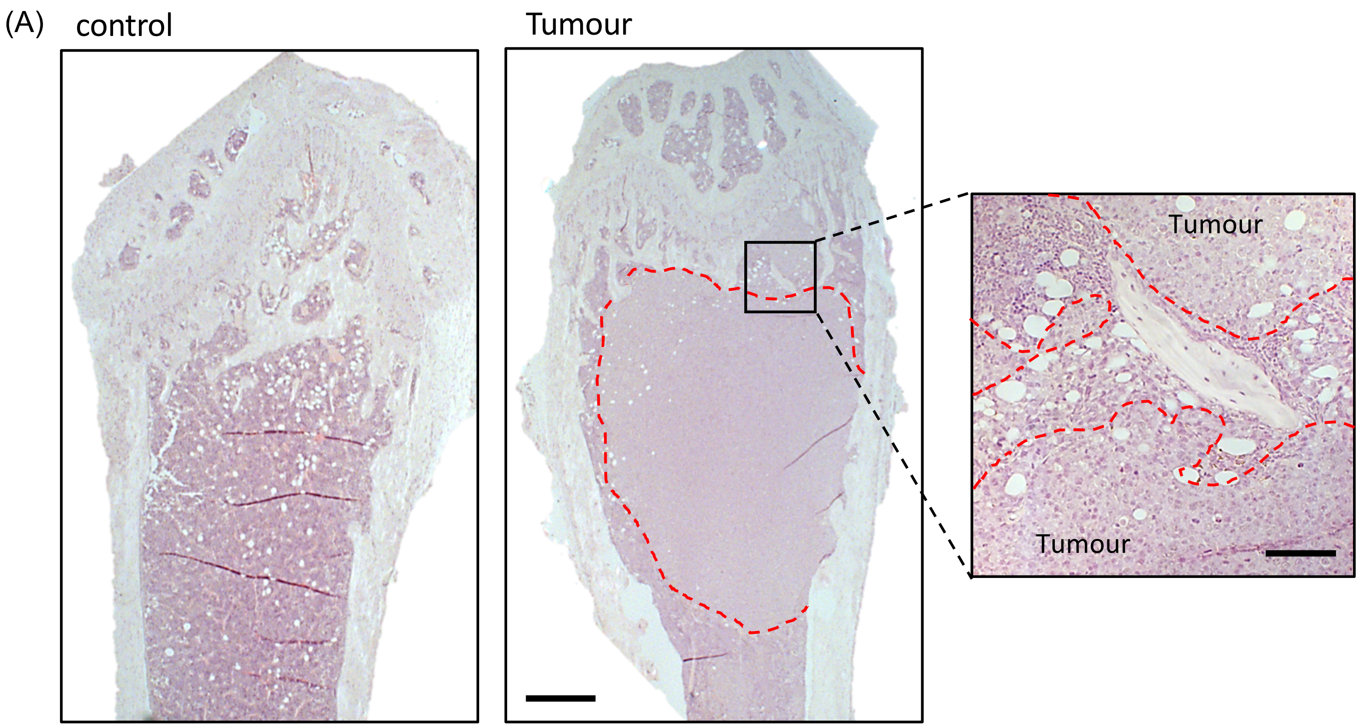

Supplemental Figure 2. Adipocytes localise to the tumour-bone interface in a human model of multiple myeloma. NOD/SCID/gamma mice were injected with JJN-3 myeloma cells and developed aggressive short-term disease. Tumour areas were identified as light patches of cells within the marrow. Tumour-bone interface is denoted by red dotted line. Scale bar 500 µm. Additional box shows a higher magnification to show the BMAds surrounding the tumour area (red dotted line denotes tumour-bone interface). Scale bar 100 µm.

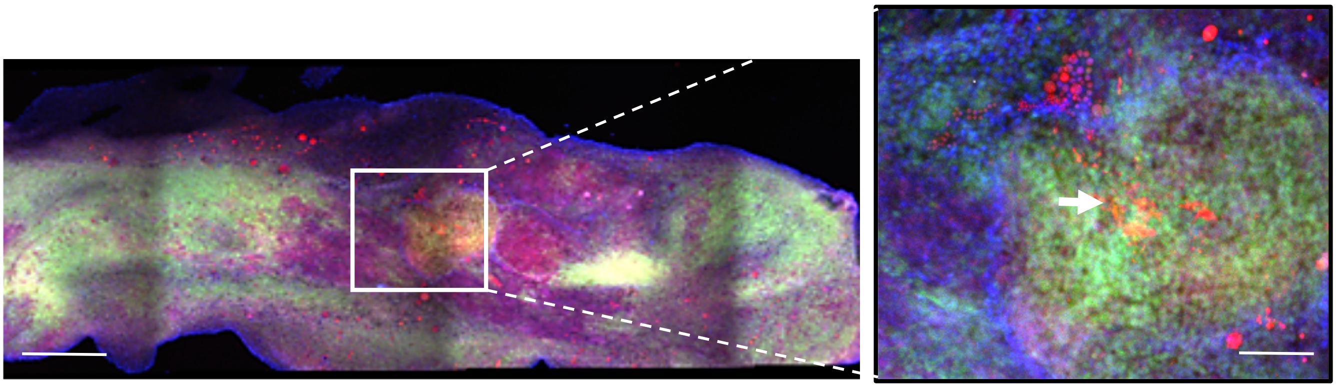

Supplemental Figure 3. 5TGM1-GFP cells uptake lipid *in vivo*. Immunofluorescence of 5TGM1-GFP (green) cells and Lipidtox (red) to visualise adipocytes/lipid. Scale bars 200 μm – 50 μm.

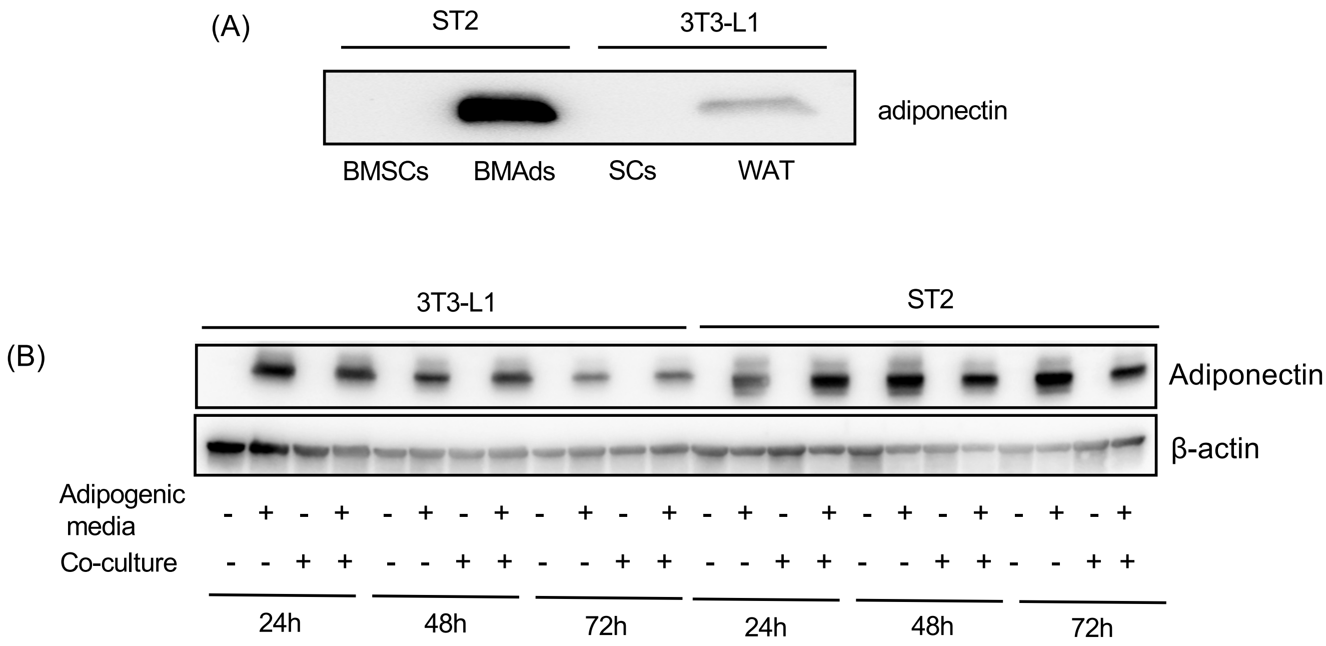

Figure 4. Myeloma cells decrease adiponectin expression in BMAds but not in WAT-like adipocytes (A) Adiponectin protein expression was measured in conditioned media taken from undifferentiated (BMSCs/SCs) and differentiated (BMAds/WAT) ST2 cells and 3T3-L1 cells using immunoblotting. **(B)** Adiponectin expression in BMSCs/SCs and BMAds/WAT cultured alone or in co-culture with 5TGM1 myeloma cells.

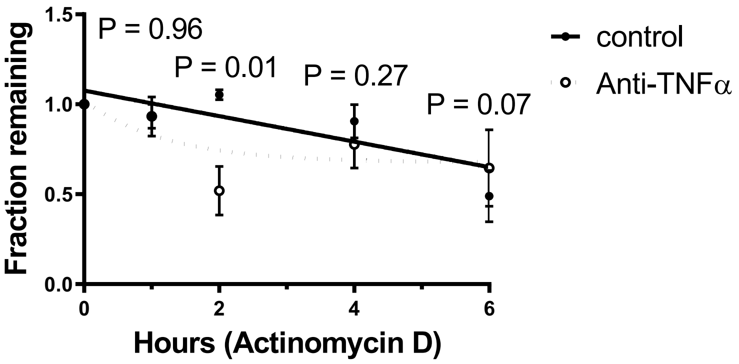

Supplemental Figure 5. TNF-α treatment causes *Adipoq* mRNA instability in ST2-derived BMAds. BMAds were treated for 24 hrs with 10ng/ml of TNF-α, *Adipoq* mRNA stability was assessed by qPCR following the addition of 10μg/ml actinomycin D for 0, 1, 2, 4 or 6 hours. Data points represent the mean (±SE) of three independent experiments. Statistical analysis is compared to 0 hrs control.

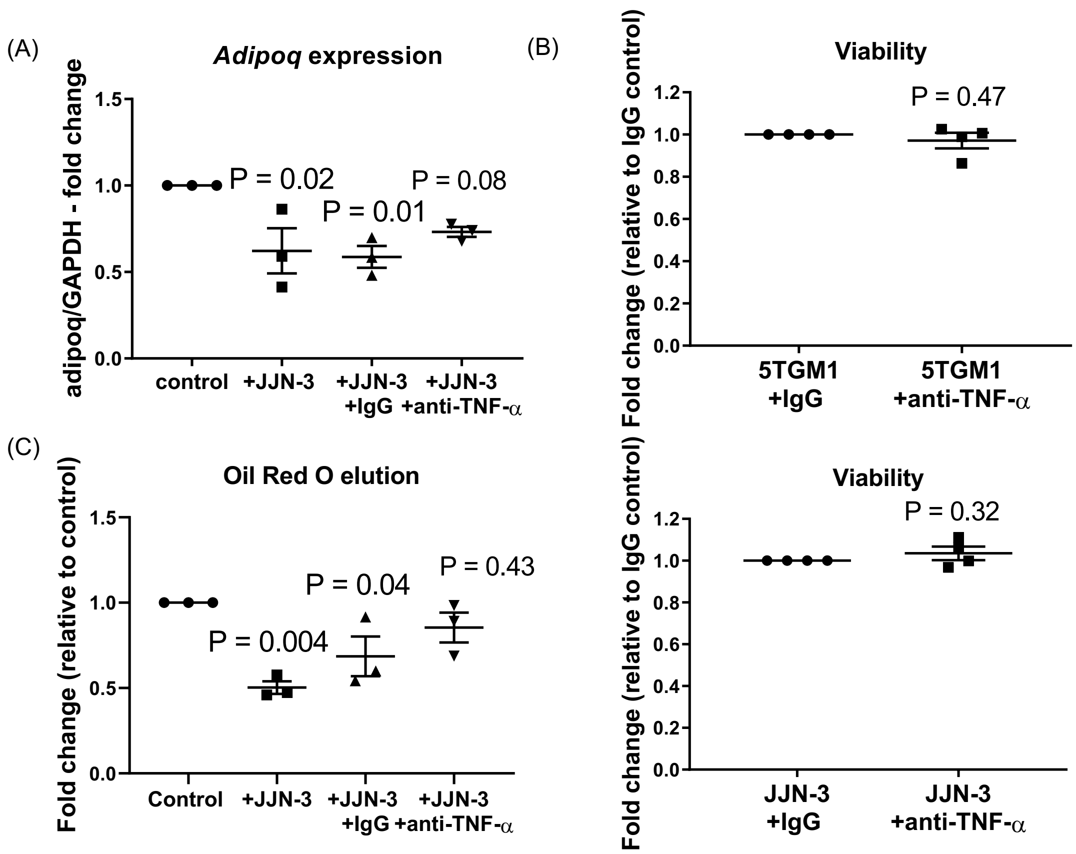

Supplemental Figure 6. TNF-α treatment decreases *Adipoq* expression. (A) BMAds were cultured with myeloma cells (JJN-3) for 48 h in the presence or absence of an anti-TNF-α neutralising antibody, *Adipoq* expression was assessed by RT-PCR. Data points represent the mean (±SE) of three independent experiments. Statistical analysis is compared to control. **(B)** Viability was measured using Alamar Blue. Results are expressed as fold change relative to IgG control. Data points represent the mean (±SE) of four independent experiments. **(C)** BMAds were cultured with myeloma cells (JJN-3) for 72 hrs in the presence or absence of an anti-TNF-α neutralising antibody, cells were fixed and stained with Oil red O. The stain was eluted and the absorbance measured. Data points represent the mean (±SE) of three independent experiments. Statistical analysis is compared to control.

Hours	Dunnett's multiple comparisons test	Adipsin (adjusted P value)	Resistin (adjusted P value)	Visfatin (adjusted P value)
24	Control vs. JJN-3	0.2303	0.9801	0.9981
24	Control vs. MM1.S	0.9913	0.6181	0.7367
24	Control vs. 5TGM1	0.3120	0.9402	0.9856
48	Control vs. JJN-3	0.2011	0.7767	0.5995
48	Control vs. MM1.S	0.5149	0.9986	0.9787
48	Control vs. 5TGM1	0.1744	0.9060	0.8862
72	Control vs. JJN-3	0.4351	0.9956	0.8950
72	Control vs. MM1.S	0.8552	0.9197	0.9997
72	Control vs. 5TGM1	0.4670	0.4469	0.7853

Supplemental Table 1. P values associated with Figure 6G. *CFD/*Adispsin, *ADSF/*Resistin and *Nampt/*Visfatin expression was assessed by RT-PCR. Data points represent the mean (±SE) of three independent experiments. Data was analysed using a 2 way ANOVA. statistical significance was calculated compared to control.
